# Combined Transcriptome and Proteome Analysis of Masson Pine (*Pinus massoniana* Lamb.) Seedling Root in Response to Nitrate and Ammonium Supplementations

**DOI:** 10.3390/ijms21207548

**Published:** 2020-10-13

**Authors:** Qifei Ren, Yunchao Zhou, Xinwei Zhou

**Affiliations:** 1 Institute for Forest Resources and Environment Research Center of Guizhou Province, Plateau Mountain Forest Cultivation Key Laboratory of Guizhou Province,College of Forestry, Guizhou University, Guiyang 550025, China; renqifei1985_2006@126.com (Q.R.); lovezxww@163.com (X.Z.); 2 Guizhou Botanical Garden, Guiyang 550004, China

**Keywords:** ammonium-N, asparagine synthetase, C/N metabolism, glutathione, nitrate-N, nitrogen preference, pine, TCA cycle

## Abstract

Nitrogen (N) is an essential nutrient for plant growth and development. Plant species respond to N fluctuations and N sources, i.e., ammonium or nitrate, differently. Masson pine (*Pinus massoniana* Lamb.) is one of the pioneer plants in the southern forests of China. It shows better growth when grown in medium containing ammonium as compared to nitrate. In this study, we had grown masson pine seedlings in medium containing ammonium, nitrate, and a mixture of both, and performed comparative transcriptome and proteome analyses to observe the differential signatures. Our transcriptome and proteome resulted in the identification of 1593 and 71 differentially expressed genes and proteins, respectively. Overall, the masson pine roots had better performance when fed with a mixture of ammonium and nitrate. The transcriptomic and proteomics results combined with the root morphological responses suggest that when ammonium is supplied as a sole N-source to masson pine seedlings, the expression of ammonium transporters and other non-specific NH_4_^+^-channels increased, resulting in higher NH_4_^+^ concentrations. This stimulates lateral roots branching as evidenced from increased number of root tips. We discussed the root performance in association with ethylene responsive transcription factors, WRKYs, and MADS-box transcription factors. The differential analysis data suggest that the adaptability of roots to ammonium is possibly through the promotion of TCA cycle, owing to the higher expression of malate synthase and malate dehydrogenase. Masson pine seedlings managed the increased NH_4_^+^ influx by rerouting N resources to asparagine production. Additionally, flavonoid biosynthesis and flavone and flavonol biosynthesis pathways were differentially regulated in response to increased ammonium influx. Finally, changes in the glutathione s-transferase genes suggested the role of glutathione cycle in scavenging the possible stress induced by excess NH_4_^+^. These results demonstrate that masson pine shows increased growth when grown under ammonium by increased N assimilation. Furthermore, it can tolerate high NH_4_^+^ content by involving asparagine biosynthesis and glutathione cycle.

## 1. Introduction

Nitrogen (N) is the building block of fundamental biological molecules and a key nutrient for plant growth and development. Because it is a major limiting nutrient in growth and development of plants, substantial amount of N is used to achieve high growth and productivity. N is taken up by plant roots directly both in organic (amino acids, peptides, and proteins) and inorganic (such as nitrate (NO_3_^−^), ammonium (NH_4_^+^), and urea) forms [1]. The two major inorganic N sources for plants, i.e., nitrate and ammonium differ in their chemical property as well as their availability in soils [2]. When present in water, both N-sources have similar diffusion coefficients but in soil, they behave differently due to negative ion charge and viscosity of the soil and other complex soil properties. Therefore, plants adapt their root morphology in response to N-source to optimize N absorption. It has been seen in earlier reports that lateral root branching is stimulated by ammonium, whereas nitrate stimulates lateral root elongation. Both N sources affect the plant differently; however, the N utilization efficiency also varies in different plant species. For example, it is known that many forest tree species utilize ammonium-N [3]. The higher diffusion constant of nitrate (10–100 times higher than ammonium) allows it to be rapidly transported towards roots [4]. On the other hand, the energy expenditure to acquire ammonium-N is far less than nitrate-N [2]. But at the same time, it has been established that ammonium accumulation in roots can cause toxicity [5,6]. Nevertheless, different plant species have demonstrated preference to ammonium, e.g., *Picea glauca* and *Pinus radiate* [6,7]. These reports suggested that relatively slower growth in nitrate rich environments was possibly due to highly atrophied transport systems for the ion. On the other hand, the rich ammonium content in the forest soils due to low nitrification potential could be a driving factor for the preference [8].

Nitrate transport, with a net negative charge, is accompanied with two protons through specific transporter, i.e., nitrate transporters (NRTs) [9,10]. Two nitrate transport systems, i.e., low-affinity transport system (LATS) and the high-affinity transport system (HATS), act coordinately to take up nitrate from soil through plant roots. Both transport systems involve NRT1 and NRT2 families, respectively [9]. Additionally, chloride channels (CLC) have also been implicated in NO_3_^−^/H^+^ accumulation in vacuoles [11]. Once, N (as nitrate) arrives in root, it is reduced to nitrite catalyzed by nitrate reductase (NR) and then further reduced to NH_4_^+^ and the reaction is catalyzed by nitrite reductase (NiR). In contrast, ammonium, with a net positive charge, is eletrogenically transported by ammonium transporters (AMTs) [12,13]. Regarding ammonium transport, physiological and ammonium influx studies have shown AMTs with high affinities are located in lateral root (like AMT1.1, AMT1.3, and AMT1.5) [14,15]. Other channels such as aquaporins (AQPs), potassium channels, and non-selective channels have also been associated with NH_4_^+^ transport [16]. Ammonium that is transported to the roots or formed by reduction of nitrite is assimilated in plastid by GS/GOGAT cycle [16]. Glutamine synthase (GS) catalyzes the formation of glutamine by fixing NH_4_^+^ on a glutamate and the glutamine reacts with 2-oxoglutarate (2OG) to produce two molecules of glutamate. Additionally, cytosolic NH_4_^+^ assimilation is carried out by asparagine synthetase (AS), where amino group of glutamine is transferred to a molecule of aspartate in a ATP-dependent manner to generate glutamate and asparagine [17,18]. Recent studies using proteomics approach in Arabidopsis have shown that (carbon) C/N metabolism is modulated when plants are fed with ammonium, and the authors suggested that this modulation is probably driven by alternative C provision routes to tricarboxylic cycle (TCA) while contributing H^+^ balance [19].

Categorizing plants for their N-source preference is an open subject, and literature is continuously being added to this regard. Earlier, Falkengren-Grerup [20] described the interspecific differences in the preference of ammonium and nitrate in 23 vascular plants. Britto and Kronzucker [21] described that N-source preference is much more complex, and sophisticated models need to be developed for its understanding. This is mainly due to interaction between both N-sources, environmental variables, i.e., temperature, soil type, soil pH, and nutrient influx and efflux in the root zone. Recent developments in genomics technologies are facilitating detailed understanding of previously unknown pathways. For example, a study on early N-deprivation in Arabidopsis root revealed that plasma membrane pH and elevated regulation of osmotic homeostasis under ammonium deprivation lead to adjustments in post-translation modifications as compared to nitrate [22]. Another study identified sets of nitrate and ammonium-specific genes. It was reported that ammonium can play a significant role as a signal too, where NH_4_^+^ assimilation related metabolites (glutamate or glutamine) can actually trigger changes in gene expression. Furthermore, it was revealed that ammonium-specific response is primarily linked with biotic stress and plant defense [23]. Nevertheless, the functionalities of ammonium in plants are not restricted since high NH_4_^+^ contents protects plants from pathogen attack, improves the cross-tolerance to other abiotic stresses, and improves the quality of crops [24].

Masson pine (*Pinus massoniana* Lamb.) is one of the important plant species in the southern forests of China [25]. It has gained the status of main pioneer species for forest/vegetation restoration in China. However, this species may not grow well in sites with N gradients because of their preference of ammonium and/or relatively higher soil nitrate availability, where the latter is more prevalent in such sites [26]. In a pilot study, we observed that masson pine seedlings grow better in ammonium as compared to nitrate in small sand culture conditions. Preference of an N source can be an important determinant and predictor of masson pine distribution and interactions with other species. In order to understand the genetic basis underlying this preference and to delineate the transcriptomic and proteomic signatures governing this preference, we have performed a combined proteomic and transcriptomic study in the roots of masson pine under three non-toxic N-conditions, i.e., pure ammonium, pure nitrate and a mixture of both. Here, we discuss the differential signatures, which help masson pine grow better in ammonium.

## 2. Results

### 2.1. Morphological Response of Pine Roots under Different N-Nutrition

**Figure 1:**
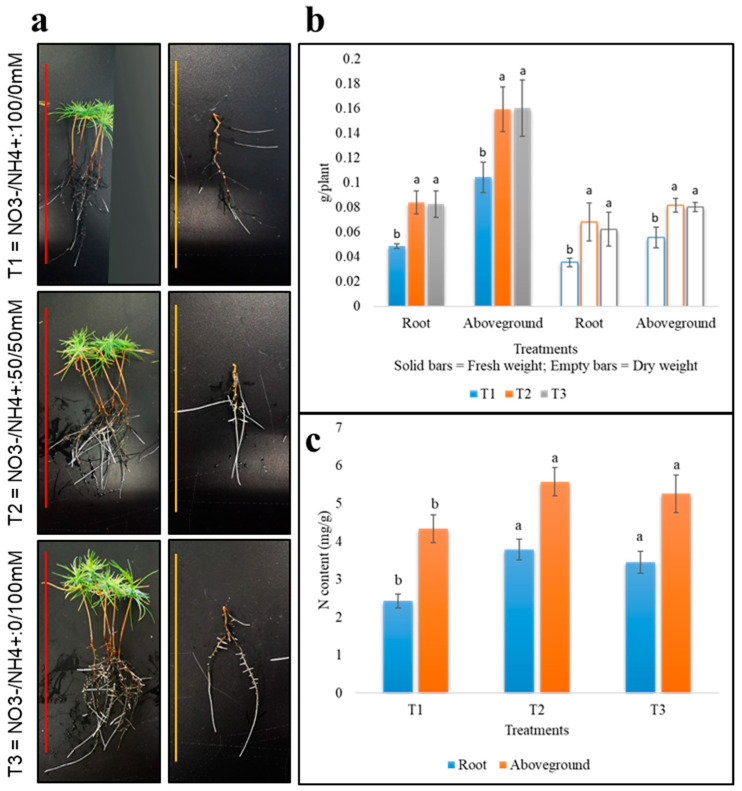
(**a**) Seedlings of *Pinus massoniana* treated with different N forms for 30 days. The red line = 20 cm, and the orange line = 15 cm. (**b**) Fresh and dry weight of roots and aboveground parts of seedlings. (**c**) Nitrogen content of roots and aboveground parts of the seedlings. Bars on the graphs show standard deviation. Different letters show that the values differ significantly between treatments.

The data on the growing masson pine seedlings in different N sources, i.e., T1: NO_3_^−^/NH_4_^+^: 100/0 mM, T2: NO_3_^−^/NH_4_^+^: 50/50 mM, and T3: NO_3_^−^/NH_4_^+^: 0/100 mM, for 30 days showed that T3 had the highest no. of root tips and longest roots. T1 had significantly lower root length, and average no. of root tips as compared to T3 (Table 1; Figure 1a). There was no significant difference for root surface area data between T2 and T3, but T1 was significantly lower than T2 and T3 for this trait. Average root volume differed significantly between T1 and T3 (Table 1; Figure 1a). Fresh and dry weights had similar pattern in aboveground part and in the roots, i.e., the weights were significantly lower in T1 as compared to T2 and T3, while weight data did not differ significantly between T2 and T3 (Figure 1b). The N content of roots and aboveground parts followed the same pattern (Figure 1c). Together, these observations suggest that when masson pine seedlings are supplied with NH_4_^+^ alone or in combination with NO_3_^−^, an improvement in the studied traits was observed.

### 2.2. RNA Sequencing and Transcript Annotation

**Figure 2:**
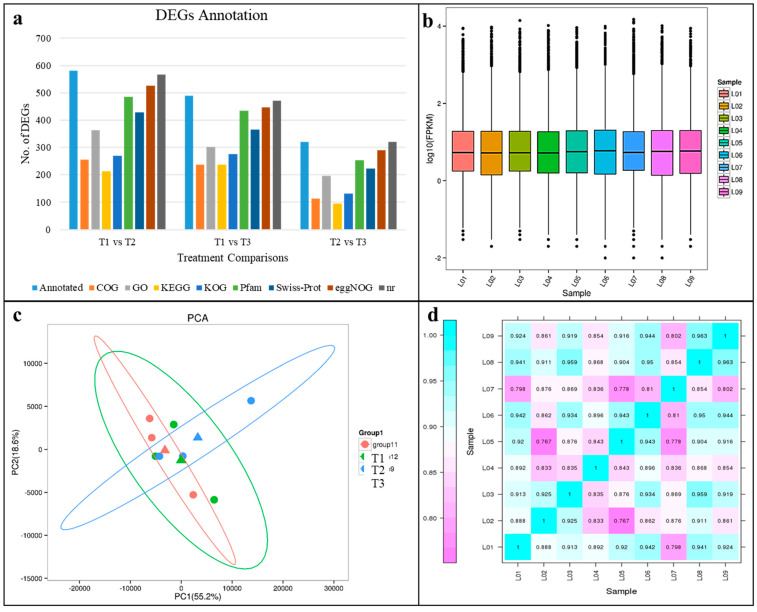
(**a**) No. of differentially expressed genes annotated in different databases, (**b**) overall distribution of sample gene expression, (**c**) principal component analysis, and (**d**) Pearson correlations between nine replicates belonging to three treatments, where T1 = L1–3, T2 = L4–5, and T3 = L7–9.

The transcriptome of the *P. massoniana* roots sequenced using Illumina Hiseq high-throughput sequencing platform showed that the reads ranging from 20.4 to 23.78 million/sample (on average 21.79 million reads) were obtained. After filtering low quality reads and adapter sequences, a total of 58.52 Gb clean data (~6.11 Gb/repeat) were obtained with Q30 based percentage ~93.13%. The clean data resulted in 34,617 unigenes, comprising 47.013 million bps with a mean length of 1358.11 bp and N50 of 2068 bp. Functional annotation of all the unigenes was conducted, a total of 6873, 10,251, 6591, 9282, 12,326, 11,304, 14,897, and 15,547 unigenes were annotated to COG, Gene ontology (GO), the Kyoto encyclopedia of genes and genomes (KEGG), eukaryotic Ortholog Groups (KOG), Pfam, Swiss-Prot, eggNOG, and non-redundant (NR), respectively. The functional DEGs annotation summary in different databases is given in Figure 2a.

The overall distribution of the gene expression based on the Fragments Per Kilobase of Transcript per Million Fragments Mapped (FPKM) method is shown in Figure 2b. The replicates of each treatment tended to group together in Principal Component Analysis (PCA) plot. The PC1 explained 55.2% variation while PC2 explained 18.6% variation (Figure 2c). Pearson correlation between replicates of all the treatments ranged from 0.767 to 0.963 (Figure 2d).

### 2.3. Differential Gene Expression Analysis 

**Figure 3:**
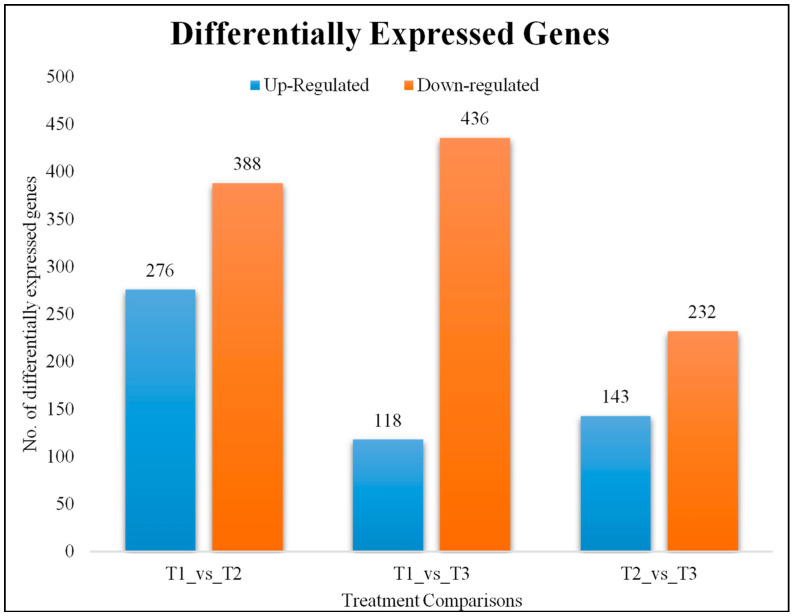
No. of differentially expressed genes (DEGs) between the tested treatment comparisons.

The screening conditions for the differentially expressed genes (DEGs) were log2 fold change ≥1 and false discovery rate (FDR) < 0.01. A total of 664, 554, and 375 DEGs were identified between T1 and T2, T1 and T3, and T2 and T3, respectively (Figure 3). We performed KEGG pathway enrichment analysis to look at the key biological pathways involved in response to different N source supplementations. The top significantly enriched pathways in T1 vs. T2 were plant hormone signal transduction, pentose and glucoronate interconversions, phenylpropanoid biosynthesis, and 62 other important pathways. In T1 vs. T3 comparison, the significantly enriched pathways were ribosome, pentose and glucoronate interconversions, carotenoid biosynthesis, and 66 other pathways. The comparison between T2 and T3 revealed the significant enrichment of phenylpropanoid biosynthesis, photosynthesis, plant-pathogen interaction, and 37 other pathways (Supplementary Figure S1).

### 2.4. DEGs Related to N Uptake and Transport

In order to understand the transcriptional responses of masson pine seedlings to the different N treatments, we searched for DEGs related to N uptake and transport in T1 vs. T2, T1 vs. T3, T2 vs. T3. Our transcriptome data showed the differential regulation of five NRTs, i.e., two (high-affinity nitrate transporters) HA-NRT 3.1s, a HA-NRT 3.2, a high affinity NRT 2.4-Like, and one NTR 1.5. The HA-NRT 3.1s were downregulated in T3 as compared to T1. Similarly, both genes were downregulated in T3 as compared to T2, which is in line with the previously established role NO_3_^−^ uptake in both conditions, i.e., when NO_3_^−^ is present alone or in presence of NH_4_^+^ [27]. The HA-NRT 2.4-like gene (*c309116*) had significantly higher expression in T1, while decreasing NO_3_^−^ resulted in decreased expression. This gene was not differentially regulated between both ammonium levels. A similar expression pattern was noticed in the case of HA-NRT 3.2. The NRT 1.5 gene (*c311742*) showed decreased expression in T2 and T3 as compared to T1. We also noticed that a CLC 1 (*c306335*) gene was expressed in both T1 and T3 where its expression reduced in T3 (log2 FC = −0.604); however, it was not differentially regulated between T1 vs. T2 and T2 vs. T3. We found that when the masson pine seedlings were supplemented with NH_4_^+^ in combination with NO_3_^−^, the expression of AMT1 (*c313513*) was increased as compared to T1 seedlings (log2FC = 0.9621). However, another transcript annotated as Rh-like/ammonium transporter (*c304554*) was downregulated in T2 as compared to T1 (log2 FC = −2.0776). Our transcriptome data showed differential regulation of five aquaporins between T1 and T2 where their expression increased in T2. One of these five aquaporins, i.e., a PIP (*c214578*) showed an interesting profile where its expression increased in T2 and T3 as compared to T1. Additionally, a sixth aquaporin (PIP2-2) was expressed in T2 and T3 and not in T1, suggesting that its expression is NH_4_^+^ specific. The expression of *c297040* (potassium channel subunit beta) doubled in T2 as compared to T1 while it did not differentially express in T3. Importantly, the expression of HAK5 (*c310320*) increased in both T2 and T3 as compared to T1, log2 FC = 1.35 and 0.98, respectively. Additionally, we found reduced expressions of CIPK2 and CIPK5 in T3 as compared to T1, increased expression of a CIPK9 in T3 over T1 and one CIPK 14-like in T3 over T2. A H^+^-transporting ATPase was differentially regulated between T2 and T3 where its expression decreased in T3 (log 2FC = −0.82) (Supplementary Tables S1–S3).

### 2.5. DEGs Related to Nitrate Reduction

As the nitrate reduction is an important step for the seedlings that were fed with nitrate, therefore, the enzymes NR, NiR, and glutamate dehydrogenases (GDH)’ expression was higher in T1 seedlings. Most importantly, the AS gene (*c308052*) was upregulated in T1 as compared to T3, while it was not differentially regulated between other two treatment comparisons, i.e., T1 vs. T2 and T2 vs. T3. Another gene (*c301526*), which was not annotated in many databases, but its molecular function (as per GO annotation) showed AS activity was also regulated in a similar manner as of the AS gene (*c308052*). These transcriptional changes suggest that nitrate is reduced to nitrite and then to ammonium in treatments fed with NO_3_^−^. Next in the mitochondria, GDH alternatively incorporates ammonium into glutamate. T1 seedlings showed increased expression of GDH (*c297315*) as compared to T3 (Supplementary Tables S1–S3).

### 2.6. DEGs Related to Carbon/Nitrogen Metabolism and Secondary Metabolism

The transcriptome comparison showed several genes related to tricarboxylic acid cycle (TCA) and secondary metabolism in seedlings treated with different N sources. We found that malate synthase (MS) was upregulated in T3 as compared to T1 and T2. Iso-citrate synthetase was upregulated in T2 as compared to T1 while downregulated in T3 as compared to T2. The acetyl-CoA synthetase was differentially regulated between T1 and T2 only; upregulated in T2 (log 2FC = 1.8878). The KEGG pathway enrichment showed that secondary metabolite (flavonoid biosynthesis) pathway is regulated. We found two dihydroflavonol 4-reductases (*c311910* and *c312351*) with contrasting expression pattern i.e., the first was upregulated in T2 as compared to T1, while the second was downregulated in T2 as compared to T1. A chalcone synthase was down regulated in T2 as compared to T1. A flavonoid 3’-monooxygenase (*c299999*) and a naringenin 3-dioxygenase were upregulated in T2 and T3 as compared to T1 (Supplementary Tables S1–S3).

### 2.7. Differentially Expressed TFs

**Figure 4:**
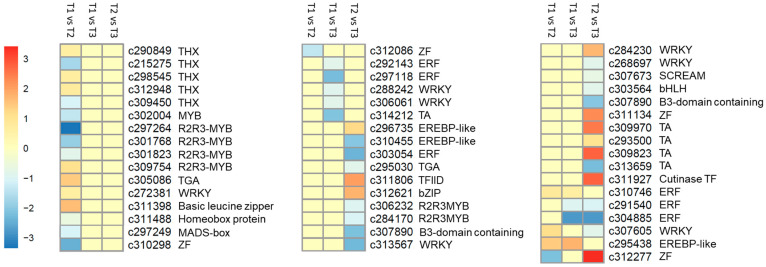
Heatmap representing log2 FC of differentially expressed transcription factors in masson pine roots treated with different N-sources. Transcript IDs are followed by the transcription factor class. TA = the transcripts that showed transcription activity but were not annotated in any database.

The differential analyses between the three treatments showed 52 TFs belonging to 19 families. Of these, 19 were differentially regulated between T1 and T2; 10 were downregulated in T2. The downregulated TFs belonged to THX (Trihelix TF), MYB (MYB-domain containing TF), HB (Homeo-box domain TF), and MADS-box TFs. Between T1 and T3, two ERFs (ethylene responsive factors), two WRKYs, and one basic TF (BTF) were regulated. All of these TFs were actually downregulated in T3 as compared to T1. The TFs that did not differentially express between T2 and T3 could be specific to NO_3_^−^ regulation. Twenty-one TFs were differentially regulated between T2 and T3; nine of these were downregulated in T3, while the rest were upregulated. These TFs could be specific to NH_4_^+^ treatment. Apart from these, we also looked for TFs that were regulated in more than one treatment comparison. Interestingly, two TFs, i.e., ERF (*c310746*) and EREBP TF (*c295438*) were upregulated in both T2 and T3 as compared to T1. These TFs could be candidates for their roles in better performance of seedlings in T2 and T3. Two ERFs (*c291540* and *c304885*) were down regulated in T3 over T1 and T2. One WRKY (*c307605*) was upregulated in T2 over T1, while it was downregulated in T3 over T2. Contrary to this, one ZnF showed downregulation in T2 as compared to T1, while upregulation in T3 as compared to T2. One bHLH (*c311080*) was downregulated both in T2 and T3 (Figure 4; Supplementary Tables S1–S4).

### 2.8. DEGs Related to Phytohormone Regulation

Fourteen DEGs were significantly enriched in plant hormone signal transduction pathway between different treatments. Of these, three were differentially expressed between T2 and T3 and their expression decreased in T3. Two genes were jasmonate ZIM domain-containing protein (*c305774* and *c313439*) and one was a TGA (TGACG-binding) TF (*c295030*). A pathogenesis related protein 6-like gene (*c309091*) was highly expressed in T1, where its expression decreased in T2 and T3. Another SAUR family protein (*c309011*) was downregulated in T3 as compared to T1. All other DEGs were only regulated between T1 and T2; six were upregulated, and three were downregulated in T2 (Table 2).

### 2.9. qRT-PCR Analyses of Selected Genes

**Figure 5:**
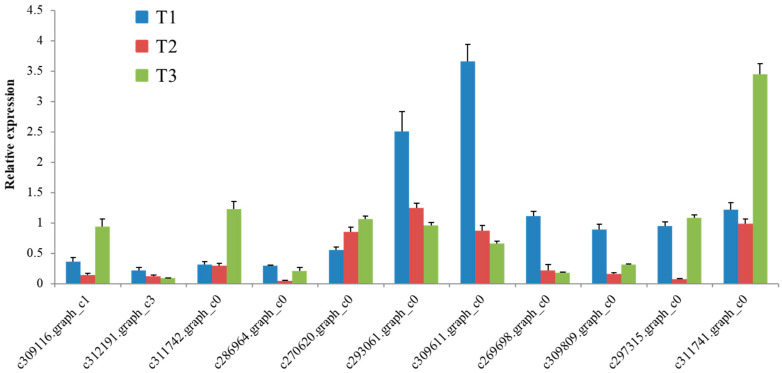
qRT-PCR validation of the selected differentially expressed genes in masson pine seedling roots grown in different nitrogen sources. The bars represent relative expression of the genes; the means were taken from three independent replications, and the error bars represent the standard deviation.

We validated the expression of eleven masson pine genes of particular interest based on their important roles in the studied pathways (Figure 5). The *tublin* (*β-TUB*) gene was used as an internal control to standardize the data, and the amount of eleven genes’ transcripts was normalized by comparing with the constitutive abundance of *β-TUB*. All the tested genes were characterized by similar expression in the RNA-seq data.

### 2.10. Proteome Analysis and Identification of DEPs

**Figure 6:**
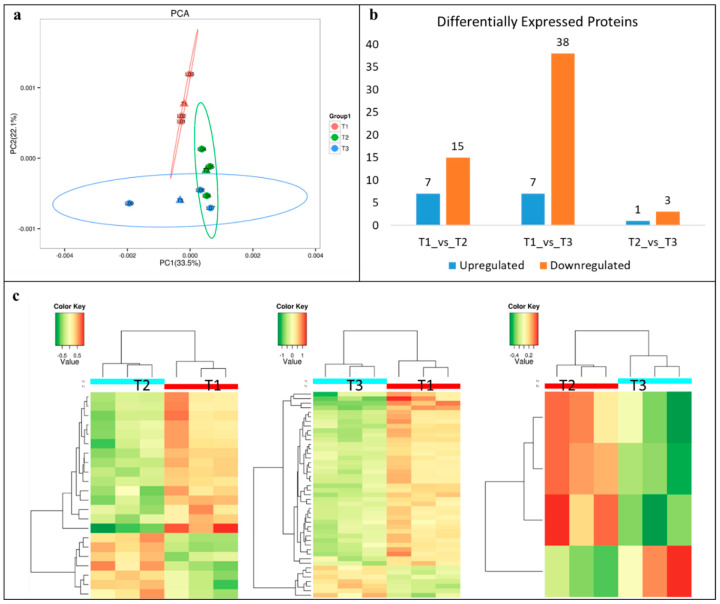
(**a**) Principal component analysis, (**b**) summary of differentially expressed proteins, and (**c**) heatmaps of the expression of the differentially expressed proteins between different treatments.

The proteome quantification of the three samples, i.e., T1, T2, and T3, resulted in the identification of 6227 proteins. Principal component analysis showed a clear separation between the treatments (Figure 6a). A total of 5284 proteins were annotated, i.e., 4845, 2127, and 3195 proteins were annotated in GO, KEGG, and COG databases, respectively. We used *t* test to analyze the differences in protein expression between the two groups of samples and screened the proteins that differed significantly (*p* < 0.01) with a fold change (FC) > 1.50. In total, 71 differentially expressed proteins (DEP) between the treatments (Figure 6b,c). A relatively higher number of DEPs were found between T1 and T3 (45) as compared to other treatment comparisons, i.e., T1 vs. T2 = 22, and T2 vs. T3 = 4 (Figure 6b,c). KEGG pathway enrichment analysis showed that inositol phosphate metabolism, glycerophospholipid metabolism, ribosome, phosphatidylinositol signaling system, and RNA degradation were significantly enriched between T1 and T2. Seventeen pathways were significantly enriched between T1 and T3. Most importantly, we found citrate cycle (TCA cycle), nitrogen metabolism, carbon metabolism, purine as well as pyrimidine metabolisms, and biosynthesis of amino acids. Nitrogen metabolism and ribosomes were the two pathways that were enriched between T2 and T3 (Supplementary Figure S2).

A protein (c309809; E.C.1.7.7.1) NiR was downregulated in T3 as compared to T1 and T2. A protein c250631 (E.C. 2.7.8.11) enriched in inositol phosphate metabolism was upregulated in T2 as compared to T1. It directly converts myo-inositol to phosphatidyl-1D-myo-inositol. The same protein was enriched in phosphatidylinositol signaling system and glycerophospholipid metabolism. The important observation was the upregulation of a protein in T3 as compared to T1 (c305505; E.C.2.3.3.1), which interconverts Acetyl-CoA to citrate and vice versa. Another protein (c309308; E.C.1.2.1.18) was also upregulated in T3 as compared to T1, which controls the interconversion of maonate semialdehyde and acetyl-CoA. Furthermore, in propanate metabolism, another protein on the downstream of E.C.1.2.1.18, i.e., c309308 (E.C.1.2.1.27) was upregulated in T3 as compared to T1. This protein is involved in conversion of (S)-Methyl-malonate semialdehyde to propanoyl-CoA, which is then converted into Acetyl-CoA through several intermediate steps (Supplementary Table S4). These observations are in accordance with the transcriptome changes.

## 3. Discussion

### 3.1. N Uptake and Root Responses to Ammonium

Our results suggest that relatively smaller number of genes, proteins, and TFs are specifically responsive to ammonium provision to masson pine roots in the experimental conditions (Figure 3). The root responses are triggered by either of the N-sources by activating different mechanisms, which are probably dependent of key molecular events. Our proteome and transcriptome approach have deepened the understanding of transcriptomic and proteomic signatures of masson pine roots supplied with either nitrate or ammonium. Higher root length and increased number of root tips under the influence of NH_4_^+^ could be because of the increased supply of ammonium due to the upregulation of the AMT1 in NH_4_^+^ fed roots (T3), since AMTs are known transporters of NH_4_^+^ [28]. However, in addition to AMTs, the increased expression of PIP (*c214578*), PIP2-2 (*c312914*), K^+^ channel subunit beta (*c297040*), and HAK5 (c310320) in T3 roots also played role in NH_4_^+^ influx. We state this because, previous studies have explained that NH_4_^+^ can also move across the plasma membrane through non-specific transport system involving potassium channels, aquaporins, and other non-selective cation channels [18,29]. We observed higher lateral root formation in NH_4_^+^ treated roots. Previous studies have elaborated the fact that ammonium stimulates lateral root branching, as obvious from our observations, and nitrate stimulates lateral root elongation, and together, both exert a local complementary effect on roots by increasing the lateral root branching and length [30]. On the other hand, we observed that the main roots in T3 are relatively shorter than T1 and T2. This is probably because of the downregulation of AGL11 in T3 as compared to T2. These observations are consistent with the previous reports that a MADS-box TF triggers larger primary roots and lateral root length as well as increases the expression of NRTs [31]. The root performance can also be due to the downregulation of the TGA TF (*c295030*) in T3 as its expression corresponds to the reduced lateral root elongation in T3 [32]. The better root morphological features in T3 as compared to T1 could also be possibly explained by the upregulation of two TFs, i.e., ERF (*c310746*) and EREBP TF (*c295438*) in both T2 and T3 as compared to T1. *c310746* is a AP2/ERF (ERF039) and *c295438* is ERF RAP2-3, as both have been implicated in ethylene responses in different abiotic stresses [33,34], but their roles in nitrogen assimilation need further exploration. Additionally, the TFs that were regulated between T2 and T3 are important candidates for further exploration. The ERF-LEP (*c296735*) that was upregulated in T3 has been reported to be involved in leaf petiole development [35], vascular cell number [36], and gibberellin-induced germination [37]. WRKY12’s (*c284230*) exclusive regulation between ammonium fed masson pine roots is in accordance with a recent report in maize where authors reported similar exclusive expression of WRKY TFs [38]. Some studies have reported the expression of WRKYs, MADS, and EREBPs in response to N deficiency [39], but the ammonium regulated functional characterization has not been reported yet.

### 3.2. Nitrogen Assimilation—The GS/GOGAT Cycle

**Figure 7:**
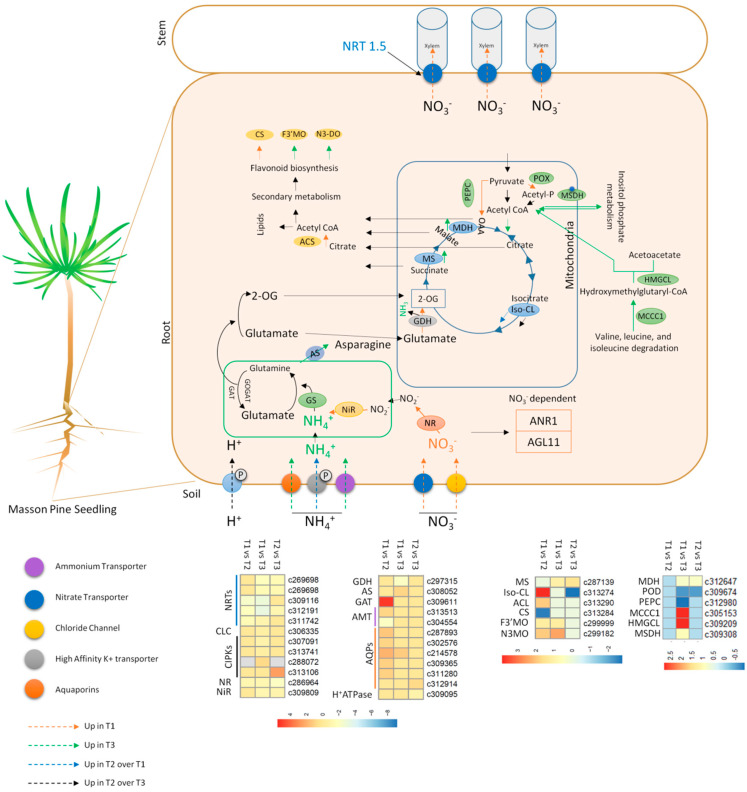
Schematic representation of key transcriptional changes in masson pine seedlings grown in nitrate (T1), mix of nitrate and ammonium (T2), and ammonium (T3). Green and blue boxes represent chloroplast and mitochondria, respectively. The colors of arrows represent their respective regulation by either of the treatment. NRTs (nitrate transporters), AMTs (ammonium transporters), CLC (chloride channel), CIPK (CBL-interacting protein kinase), NR (nitrate reductase), NiR (nitrite reductase), GS (glutamine synthetase), GOGAT (glutamate synthase), AS (asparagine synthetase), GDH (glutamate dehydrogenase), 2-OG (2-oxoglutarate), MS (malate synthase), MDH (malate dehydrogenase) OAA (oxaloacetate), Iso-CL (isocitrate lyase), AQP (aquaporins), ACL (ATP citrate lyase), CS (citrate synthase), F3’MO (flavonoid 3’-monooxygenase), N3DO (naringenin 3-dioxygenase), PEPC (phosphoenolpyruvate carboxylase), POX (pyruvate oxidase), MSDH (malonate-semialdehyde dehydrogenase), HMGL (methylmalonate-semialdehyde dehydrogenase), MCCC1 (3-methylcrotonyl-CoA carboxylase alpha subunit). The heatmaps show the log2 foldchange values (in three treatment comparisons) of differentially expressed genes in *P. massoniana* roots.

Upon direct absorption in the roots (or by conversion of NO_2_^−^ by the action of NiR), the NH_4_^+^ takes part in the glutamate to glutamine inter-conversion reaction by the GS/GOGAT enzyme system [40]. Our transcriptome and proteome data did not show differential expression of the GOGAT or GS enzyme. This suggests that the nitrogen assimilation process at this stage worked similarly regardless of the N-source (Supplementary Tables S1–S4). Since in T1, the masson pine roots had to convert the incoming NO_3_^−^ into NH_4_^+^, the NR genes (which converts NO_3_^−^ to NO_2_^−^) was upregulated in T1 as compared to T3. For the next step, i.e., NO_2_^−^ to NH_4_^+^, the required enzyme NiR was upregulated in T1 as compared to T3, which is evident from transcriptome and proteome data (Figure 7; Supplementary Table S4) [2]. These observations are quite similar to the recent work on the adaption of Arabidopsis roots to ammonium [19]. These observations, as supported by previous study, suggest that masson pine seedling roots exhibit no differential regulation of GS/COGAT when fed with either of the N-sources. The only enzyme GDH (probable NADP-dependent GDH) was upregulated in T1 as compared to T3 (Supplementary Table S2). This is different from the study on Arabidopsis, where the authors did not find its differential regulation at proteome scale [19]. Similarly, we also did not find GDH differential expression in our proteome data. It is also known that the expression of GDH is affected by a number of different factors such as C shortage, dark-adaptation, sucrose starvation, and biotic and abiotic stresses [41,42]. Based on the GDH expression observed in T1, we deduced that when fed with nitrate, masson pine seedlings might employ GDH to sustain growth under low C/N ratios. Contrarily, the ammonium fed masson pine seedlings could have adopted the asparagine synthesis [43], which is discussed in next section.

### 3.3. Carbon/Nitrogen Metabolism Modulation in Response to NH_4_^+^ and the Role of TCA Cycle

Plants’ ability to grow better and produce higher biomass is largely dependent on N supplementation and its balance with carbon. The C/N balance in plants is regulated by the availability of C skeletons, energy sources, and important biomolecules for N assimilation related pathways [44]. With the increased uptake (supply) of NH_4_^+^, plants will need to induce pathways to assimilate the received/produced NH_4_^+^, which in turn requires increased energy and C [19]. We found that DEGs and DEPs related to TCA cycle, i.e., MS and MD, were abundant in T3 as well as T2, clearly indicating that under ammonium nutrition, masson pine root adapts by the promotion of TCA flux mode to sustain C skeleton availability (Figure 7). This in turn leads to effective NH_4_^+^ optimal storage in asparagine since we also found the upregulation of gene related to L-asparagine metabolism, i.e., AS (*c308052*) (Supplementary Tables S2 and S3). A similar mechanism was revealed in wheat, where isotopic labelling showed the efficient adaptation of wheat root TCA cycle flux modes in order to match the carbon demand under ammonium nutrition [45]. This is further supported by the upregulation of the DEP c309308 (malonate-semialdehyde dehydrogenase). This enzyme converts malonic semialdehyde to acetyl-CoA (in inositol phosphate metabolism), thus increasing acetyl-CoA reserves to be further converted to citrate by c305505 (upregulated in T3). Thus, our results suggest that masson pine roots adapt to ammonium nutrition by increased N assimilation through the promotion of TCA flux modes.

### 3.4. Managing Increased NH_4_^+^ Accumulation in Masson Pine Roots

Increased ammonium influx has been largely considered as a universal stressful condition for plant [24]. The plant genotypes or species that withstand increased NH_4_^+^ accumulation show tolerance in different ways. Since, in soils, presence of NH_4_^+^ as a sole N source does not exist; however, certain natural ecosystem factors and the use of nitrification inhibitors lead towards increased stable and higher NH_4_^+^ content [24]. To avoid, NH_4_^+^ stress, plants adapt various strategies to protect their cells from NH_4_^+^ induced stress such as higher level of free asparagine due to its important role in protecting the cell [46]. Our observations that T3 plants had increased expression of ASs indicate that masson pine roots sense higher NH_4_^+^ levels (Figure 7; Supplementary Table S3). Since, there is a consensus that AS expression is induced by a reduction in soluble carbohydrate supply or dark [47]. We found that in T3, as compared to T1, a beta-glucosidase 12-like gene (glycosyl hydrolase family 1 member; *c291550*) was downregulated (Supplementary Table S3). This proposition is also based on the fact that in T1 vs. T3, 14 other genes related to carbohydrate transport and metabolism were downregulated in T3 (see DEGs in Supplementary Table S2 that are highlighted in yellow background). Most importantly, the downregulated DEGs were, i.e., pectate lyases (c312286, c293839, c309368, and c312142), pyruvate decarboxylase 4 (c285412), polygalacturonase (c307368), chitinase like protein 1 (c295894), endoglucanase (c306748), linamarin synthase (c307027), 6-phosphogluconate dehydrogenase (c283906), and galactinol-sucrose galactosyltransferase (c310284). Since, these genes are important part of carbohydrate transport and metabolism and affect (directly or indirectly) the carbohydrate supply [48,49,50,51,52,53,54]; therefore, the reduced expression lead the reduced carbohydrate supply and may have induced the AS expression. Another inducer of AS’s expression is the increased supply of reduced nitrogen, i.e., NH_4_^+^ itself [55,56]. Hence, it could be stated that masson pine seedlings, when grown solely in NH_4_^+^, had the ability to tolerate increased NH_4_^+^ supplies by increasing the expression of AS. Furthermore, it is known that N can be redirected from glutamine to asparagine when excess NH_4_^+^ is available [38].

The regulation of flavonoid biosynthesis and flavone and flavonol biosynthesis pathways between T1 and T3 clearly indicated that these pathways respond to sole NH_4_^+^ supplies (Supplementary Figure S2). However, limited information is available about role of secondary metabolites in NH_4_^+^ stress, and all the reports suggested their involvement in defense against pathogens and nutrition improvement [see review by [24]]. Whether the upregulation of flavonoid 3’-monooxygenase and naringenin 3-dioxygenase is just a consequence of ammonium assimilation increase or if they have a dedicated role in ammonium stress is a question for further elucidation.

Another common NH_4_^+^ response in plants is the glutathione metabolism, which induces antioxidant machinery and provides resistance to different abiotic stress, e.g., salt stress [57]. The upregulation of glutathione s-transferases in T2 (*c291486*, *c307711*, *c292057*) and in T3 (*c298941*) as compared to T1 indicates that ammonium provision solely or in combination with nitrate triggered glutathione s-transferase activity (Supplementary Tables S1 and S2). Studies have reported that internal ammonium excess induces reactive oxygen species (ROS) bursts [58]. Because it is known that glutathione s-transferases catalyze the transfer of superoxide free radicals to reductive glutathione, which results in the detoxification of the oxidants [59]. Therefore, changes in expression levels of glutathione s-transferases indicate that glutathione cycle has strong role in scavenging the NH_4_^+^ excess induced ROS as observed in rice [58].

Finally, we noticed the limited changes in the signaling of phytohormones in response to ammonium as compared to nitrate; however, a relatively larger number of DEGs were regulated between T1 and T2 (Table 2). The downregulation of a SAUR protein in NH_4_^+^ fed plants is consistent with the previously observations in nitrogen-deprived Arabidopsis roots [22]. The relationship of its expression with ammonium is yet to be explored; however, it is well established that auxin signaling, transport, and transduction are significantly altered by nitrate [60]. Therefore, it could be stated that the expression of c309011 (SAUR family protein) in T1 could be driven by nitrate. We state this because we also found the upregulation of other SAUR family members in T1 (Table 2). A dedicated study on the role of either of the N-sources on phytohormone biosynthesis and signaling in relation to the changes in root system architecture would increase the understanding the signaling pathways related to N-source based hormonal changes.

## 4. Material and Methods

### 4.1. Plant Material and Treatments

Masson pine seeds (*P. massoniana* Lamb) were collected from the excellent half-sib family individual plants of the *Pinus* national seed base in Ma’anshan, Duyun City, Guizhou Province, in January 2020. The seeds were sterilized with 0.5% potassium permanganate solution for 1 h, rinsed with sterile water 3~5 times, and immersed in sterile water with an initial temperature of 4 °C for 24 h. Seeds were germinated, in a germination box containing vermiculite and perlite, in an artificial climate box (day/night = 14 h/10 h and temperature 24/22 °C). Sixty days old seedlings were pre-cultured in a 1:1 solution of NH_4_^+^: NO_3_^−^ for 3 days, and then, the seedlings were placed in N-free water to adapt for 1 week prior to experimental treatments, i.e., T1: NO_3_^−^/NH_4_^+^: 100/0 mM, T2: NO_3_^−^/NH_4_^+^: 50/50 mM, and T3: NO_3_^−^/NH_4_^+^: 0/100 mM, for 30 days. Nutrient solution was changed every three days and the pH (5.5–5.6) was adjusted twice a day (9 am and 5 pm). The concentration of total N (1 mm/L) and the ion balance in the three treatments were maintained as given in the Table 3. We added nitrification inhibitor 7 µmol/L dicyandiamide in the medium to prevent the conversion of NH_4_^+^ to NO_3_^−^. The experiment was conducted in a triplicate completely randomized design. Samples, three biological replicates, were taken from the roots and stored at −80 °C for RNA extraction or used for determination of growth index.

### 4.2. Morphological Evaluation and N-Content Measurements

The seedlings were rinsed with excess amount of tap water followed by deionized water (thrice) before the measurement of root characteristics. Root lengths were measured with a ruler. Fresh weight was determined by removing excess water on an absorbent paper and then weighing on an electronic balance (PCE-ABT 220, ±0.5 mg accuracy). Roots were oven dried at 105 °C for 30 min and then at a constant temperature, i.e., 70 °C for 48 h before dry weight was measured. Roots were scanned and analyzed with Epson digital scanner and Win RHIZO software, and the root volume and root surface area were measured. Root N content was measured by following single sulphuric acid—hydrogen peroxide digest method by Lowther [61].

### 4.3. Library Preparation for Transcriptome Sequencing

A total amount of 1 μg RNA per sample was used as input material for the RNA sample preparations. Sequencing libraries were generated using NEBNext^®^Ultra™ RNA Library Prep Kit for Illumina^®^ (NEB, Ipswich, MA, USA) following manufacturer’s recommendations, and index codes were added to attribute sequences to each sample. Briefly, mRNA was purified from total RNA using poly-T oligo-attached magnetic beads. Fragmentation was carried out using divalent cations under elevated temperature in NEBNext First Strand Synthesis Reaction Buffer (5X). First strand cDNA was synthesized using random hexamer primer and M-MuLV Reverse Transcriptase. Second strand cDNA synthesis was subsequently performed using DNA Polymerase I and RNase H. Remaining overhangs were converted into blunt ends via exonuclease/polymerase activities. After adenylation of 3’ ends of DNA fragments, NEBNext Adaptor with hairpin loop structure was ligated to prepare for hybridization. In order to select cDNA fragments of preferentially 240 bp in length, the library fragments were purified with AMPure XP system (Beckman Coulter, Beverly, CA, USA). Then, 3 μL USER Enzyme (NEB, Ipswich, MA, USA) was used with size-selected, adaptor-ligated cDNA at 37 °C for 15 min followed by 5 min at 95 °C before PCR. Then PCR was performed with Phusion High-Fidelity DNA polymerase, Universal PCR primers, and Index (X) Primer. Finally, PCR products were purified (AMPure XP system), and library quality was assessed on the Agilent Bioanalyzer 2100 system (Beverly, CA, USA).

### 4.4. Clustering and Sequencing

The clustering of the index-coded samples was performed on a cBot Cluster Generation System using TruSeq PE Cluster Kit v3-cBot-HS (Illumina, San Diego, CA, USA) according to the manufacturer’s instructions. After cluster generation, the library preparations were sequenced on an Illumina Hiseq 2000 platform and paired-end reads were generated.

### 4.5. Data Analysis

Raw data (raw reads) of Fastq format were firstly processed through in-house perl scripts. In this step, clean data (clean reads) were obtained by removing reads containing adapter, reads containing ploy-N, and low-quality reads from raw data. At the same time, Q20, Q30, GC-content, and sequence duplication level of the clean data were calculated. All the downstream analyses were based on clean data with high quality.

### 4.6. Transcriptome Assembly

The read1 files from all libraries/samples were pooled into one big left.fq file, and read2 files into one big file. Transcriptome assembly was accomplished based on the read 1 and read 2 files using Trinity [62] with min_kmer_cov set to 2 by default and all other parameters were set to default settings.

### 4.7. Gene Functional Annotation and Differential Expression Analysis

Gene function was annotated based on the following databases: NR (NCBI non-redundant protein sequences); Pfam (Protein family); KOG/COG/eggNOG (Clusters of Orthologous Groups of proteins); Swiss-Prot; KEGG (Kyoto Encyclopedia of Genes and Genomes); GO (Gene Ontology) [63,64,65,66,67].

Gene expression levels were estimated by RSEM [68] for each sample. Clean data were mapped back onto the assembled transcriptome and the read count for each gene was obtained from the mapping results. Differential expression analysis between treatments was performed using the DESeq R package (1.10.1). The resulting P-values were adjusted using the Benjamini and Hochberg’s approach for controlling the false discovery rate [69]. Genes with an adjusted *p*-value < 0.05 found by DESeq were assigned as differentially expressed. For KEGG enrichment analyses we used KOBAS [70] software to test the statistical enrichment of differential expression genes in KEGG pathways.

### 4.8. Proteome Sequencing

#### 4.8.1. Sample Preparation, Protein Quantification, and SDS Page

Root samples stored at −80 °C were ground with liquid nitrogen and 100 mg of freeze-dried powder was taken. Added 800 μL SDT protein lysate (4% SDS, 100 mM Tris-HCl, 100 mM DTT, pH 7.6) into the 1.5 mL centrifuge tube. The mixture was put in 100 °C boiling water bath for 5 min, then in ice bath ultrasound for 10 min (100 W for 5 s with an interval of 10 s). The mixture was again put in 100 °C boiling water bath for 5 min followed by centrifugation at 14,000× *g* for 30 min. Supernatant was taken and filtered with a 0.22 μm ultrafiltration tube. We took 1 μL of each sample for BCA (bicinchoninic acid) quantification and based on the quantitative results, a 20 μg protein sample was taken for SDS-PAGE electrophoresis. The remaining samples were divided into 300 μg portions and stored in the refrigerator at −80 °C.

#### 4.8.2. Enzymatic Hydrolysis

We took 300 μg of each sample for FASP enzymatic hydrolysis and mixed the same amount of protein from each sample into an internal standard IS for enzymatic hydrolysis. Then added 200 μL UA to the sample and mixed well with buffer (8M Urea, 150 mM Tris-HCl pH 8.5), centrifuged at 14,000× *g* for 30 min at room temperature, discarded the filtrate, and repeated thrice. Then, we added 100 μL IAA (50 mM IAA in UA), shacked at 600 rpm and mixed for 1 min followed by an incubation at 300 rpm for 30 min at room temperature in dark. We then added 100 μL UA buffer, centrifuged at 14,000× *g* for 30 min at room temperature, and repeated the steps thrice. After that, we added 100 μL 25 mM/L DS buffer followed by centrifugation at 14,000× *g* for 30 min at room temperature, and this was repeated 3 times. Finally, we discarded the filtrate and added 40 μL Trypsin buffer (6 μg Trypsin in 40 μL DS buffer), placed it on a thermo-mixer (300 rpm, 18 h, 37 °C) followed by centrifugation at 14,000× *g* for 30 min at room temperature to collect the filtrate in a new collection tube. Then, 40 μL DS was added followed by centrifugation at 14,000× *g* for 30 min at room temperature; we took the filtrate and measured the optical density at 280 nm.

#### 4.8.3. Classification of Peptide Markers and LC-MS/MS Analysis

Of each group of samples, 100 μg were taken and processed according to instruction manual of TMT6plex™ Isobaric Label Reagent Set (Thermo Scientific, Waltham, MA, USA). All the labelled peptides were mixed separately for HpH pre-fractionation; Column: Gemini-NX 4.6 × 150 mm column (3 µm, 110 Å) (Phenomenex, 00F-4453-E0, Torrance, CA, USA), Buffer: Buffer A was 10 mM Ammonium acetate pH 10.0; Buffer B was 10 mM Ammonium acetate, 90% ACN, pH 10.0 on 1100 Series HPLC Value System (Agilent, Santa Clara, CA, USA). After HpH classification, each set of markers collects 40 fractions of flow-through and elution, according to The HpH chromatograms were combined into 15 aliquots and stored at −80 °C after lyophilization. 

Digested peptide samples were analyzed by LC-MS/MS on a nanoflow Easy-nLC1000 HPLC system (Thermo Scientific) connected to a quadrupole Orbitrap hybrid mass spectrometer (Q Exactive Plus, Thermo Scientific). Peptides were sprayed directly into the mass spectrometer after elution from a 75 μm × 50 cm C18 analytical column (PepMan, Thermo Scientific) on a linear gradient from 4 to 64% acetonitrile over 90 min. Proteins were identified by MS/MS using information-dependent acquisition of fragmentation spectra of multiple charged peptides. Up to 20 data-dependent fragment spectra were acquired in the linear ion trap for each full-scan spectrum at a scanning range of precursor ion of 350–2000 m/z, resolution of 60,000 at 2 m/z. The normalized collision energy was set to 35 eV. Protein identification was conducted in the built-in software SEQUEST Proteome Discoverer 2.1 (Thermo Scientific). Proteome Discoverer 2.1 performs FDR based on peptide identification results ≤ 0.01 (High Confident) for screening and quantitative analysis based on the peptide peak intensity value. The original quantitative result of protein is identification. The median of peptide quantification results was corrected based on the sum of the reported ion peak intensity values of all channels. The final quantitative results were then the ratio of the mean value of all channel intensity values, and the median of the ratio are normalized.

Functional classification of the differentially abundant proteins was carried out according to MapMan software (http://mapman.gabipd.org/es/mapman, version 3.6.0) [33]. Gene ontology (GO) enrichment analysis and visualization for cellular component and biological process were performed with BioMaps tool of VirtualPlant 1.3.

### 4.9. Gene Expression Data Validation by qRT-PCR

The primers used for qRT-PCR validation are listed in Table 4. qRT-PCR was performed using the ABI7500 Real Time System (Applied Biosystems, Foster City, CA, USA). Gene expression was analyzed quantitatively using the SYBR Green detection system with melting curve. Amplification conditions were 95 °C for 3 min, followed by 40 cycles of: denaturation, 95 °C for 15 s; annealing (55–60 °C) for 20 s; extension at 72 °C for 34 s. Samples for qRT-PCR were run in three biological replicates and two technical replicates. The results were normalized using the Pfaffl method to report relative expression [71]. For normalization of gene expression, *tubulin* (*β-TUB*) was used as internal standard.

## 5. Conclusions

Masson pine seedling roots grown in different N sources shown that ammonium-N increases overall performance of roots. The transcriptome and proteome sequencing results suggested that the expression of AMTs is increased while NRTs’ expression decreased in NH_4_^+^ grown seedlings. The increased NH_4_^+^ influx was due to regulation of many specific and non-specific NH_4_^+^ channels. We concluded that masson pine seedling possibly manages the increased NH_4_^+^ influx by promoting TCA cycle to sustain C skeleton availability and optimal NH_4_^+^ storage. Further experimentation on gene specific roles of the DEGs highlighted in our report will enable complete role of the TCA cycle in NH_4_^+^ management. Additionally, the increased NH_4_^+^ concentrations in the roots by increasing the expression of AS and by redirecting N from glutamine to asparagine. Finally, our data show that glutathione S- transferase activity is triggered under NH_4_^+^ higher supplies. This increased expression of glutathione S- transferase was probably to scavenge the NH_4_^+^ excess induced ROS.

## Figures and Tables

**Table 1:** Root morphological characteristics of masson pine seedlings treated with different N-sources, i.e., T1, T2, and T3.

	Average No. of Root Tips	Primary Root Length (cm)	Total Root Length (cm)	Root Surface Area (cm^2^)	Root Volume (cm^3^)
T1	9.2 ± 2.1 c	9.06 ± 1.71 b	33.12 ± 2.15 b	4.42 ± 0.49 b	0.065 ± 0.013 b
T2	12.5 ± 1.8 b	10.6 ± 1.39 ab	38.1 ± 1.99 a	5.44 ± 0.54 a	0.097 ± 0.013 ab
T3	18 ± 3.0 a	12.4 ± 1.62 a	39.67 ± 1.95 a	5.3 ± 0.98 a	0.077 ± 0.02 a

Values are the means followed by standard deviation. Different letters show that the values differ significantly between treatments.

**Table 2:** Differentially regulated genes/transcription factors related to phytohormone regulation.

Gene ID	log2FC			KEGG_Annotation
	T1 vs. T2	T1 vs. T3	T2 vs. T3	
*c269432*	0.731	---	---	abscisic acid receptor PYR/PYL family
*c309091*	−1.261	−1.104	---	pathogenesis-related protein 1
*c304520*	1.059	---	---	abscisic acid receptor PYR/PYL family
*c312413*	1.222	---	---	auxin-responsive protein SAUR32-like
*c305519*	1.301	---	---	bZIP; transcription factor TGA2
*c299206*	1.431	---	---	F-box protein GID2
*c306724*	1.574	---	---	SAUR family protein
*c301864*	−0.768	---	---	protein phosphatase 2C
*c272209*	−2.702	---	---	jasmonate ZIM domain-containing protein (TIFY 9)
*c294828*	2.119	---	---	SAUR-like auxin-responsive family protein
*c309011*	---	−1.981	---	SAUR family protein
*c305774*	---	---	−1.163	jasmonate ZIM domain-containing protein (TIFY 10B-like)
*c295030*	---	---	−0.831	transcription factor HBP-1b (c38)-like
*c313439*	---	---	−2.311	jasmonate ZIM domain-containing protein

--- means corresponding gene was not differentially expressed.

**Table 3:** Composition of the nutrient solution in the three treatments.

	T1	T2	T3
	NO_3_^−^/NH_4_^+^:100/0 mM	NO_3_^−^/NH_4_^+^:50/50 mM	NO_3_^−^/NH_4_^+^:0/100 mM
KNO_3_	0.6	0.1	0
Ca(NO_3_)_2_·H_2_O	0.2	0.2	0
CaCl_2_	0.425	0.425	0.625
(NH_4_)_2_SO_4_	0	0.25	0.5
KH_2_PO_4_	0.125	0.125	0.125
MgSO_4_·7H_2_O	0.25	0.25	0.25
KCl	0.15	0.65	0.75
K_2_SO_4_	0	0	0
DCD	0.007 µM/L		

**Table 4:** Primers used for qRT-PCR analysis of selected genes.

Gene ID	Gene Description	Forward Primer Sequence	Reverse Primer Sequence
*c309116*	high affinity nitrate transporter 2.4-like *(HA-NRT2.4-Like)*	GCGTTGCCTATGTCCT	TAACTGATTTCGGCTTTG
*c312191*	High-affinity nitrate transporter 3.2 *(HA-NRT3.2)*	CCATTGTATGCCTCTT	GCCTTGCTCTGATTTA
*c311742*	Nitrate transporter 1.5 (*NRT1.5*)	GATCGCTTCTACTTGTTATT	TGAGCCAGTTCTTCGT
*c286964*	nitrate reductase (NADH] (*NR*)	GACTTGATCGTCTTTCG	CAACACCGTGCCTACT
*c270620*	vacuolar amino acid transporter 1 (*AVT1C*)	TGGGACTAAGGGTCGA	TAAATCTTTCAGGCATACAG
*c293061*	Protein GLUTAMINE DUMPER 2 (*GDU2*)	ACAACCCTATGTCTCG	CAGCCATTTATCACTAT
*c309611*	Putative glutamine amidotransferase (*GAT1_2.1*)	CATGCGTTCAGGGTGG	CTATCATCATCAGGGCGTAA
*c269698*	High-affinity nitrate transporter 3.1 (*HA-NRT3.1*)	TAGCCACAGAATCCTATCAA	GGGCAGAGCACCAACA
*c309809*	ferredoxin-nitrite reductase (*NiR*)	GCAAGAGCCCTGAAAA	ACCGAAGGCAGTAGCA
*c297315*	glutamate dehydrogenase (NAD(P)+) (*GDH*)	AAACAGATGCGGGATA	AAGGTCGGATACAACG
*c311741*	peptide-N4-(N-acetyl-beta-glucosaminyl) asparagine amidase (*PNGase A*)	AGACCTCGCAGGCAGT	GATTCATCCGCAAACAC
*tuublin β-TUB*	Internal standard	GTCGTGAATCATGGCATGGC	GCCTCACTATCGGTTTCCCA

## Supplementary Materials

The following are available online at https://www.mdpi.com/1422-0067/21/20/7548/s1. Table S1: Differentially expressed genes between T1 and T2. Table S2: Differentially expressed genes between T1 and T3. Table S3: Differentially Expressed genes between T2 and T3. Table S4: Differentially expressed proteins in masson pine roots treated with different nitrogen sources. Figure S1: KEGG pathway enrichment based on differentially expressed genes between treatments. Figure S2: KEGG pathway enrichment based on differentially expressed proteins between treatments.
