# Supplementary material for: Combined Transcriptome and Proteome Analysis of Masson Pine (Pinus massoniana Lamb.) Seedling Root in Response to Nitrate and Ammonium Supplementations
Source: Int J Mol Sci. 2020 Oct 13;21(20):7548. doi: 10.3390/ijms21207548 (PMC7593940; doi:10.3390/ijms21207548)

**Supplementary Figure 1.** KEGG pathway enrichment based on differentially expressed genes between treatments.


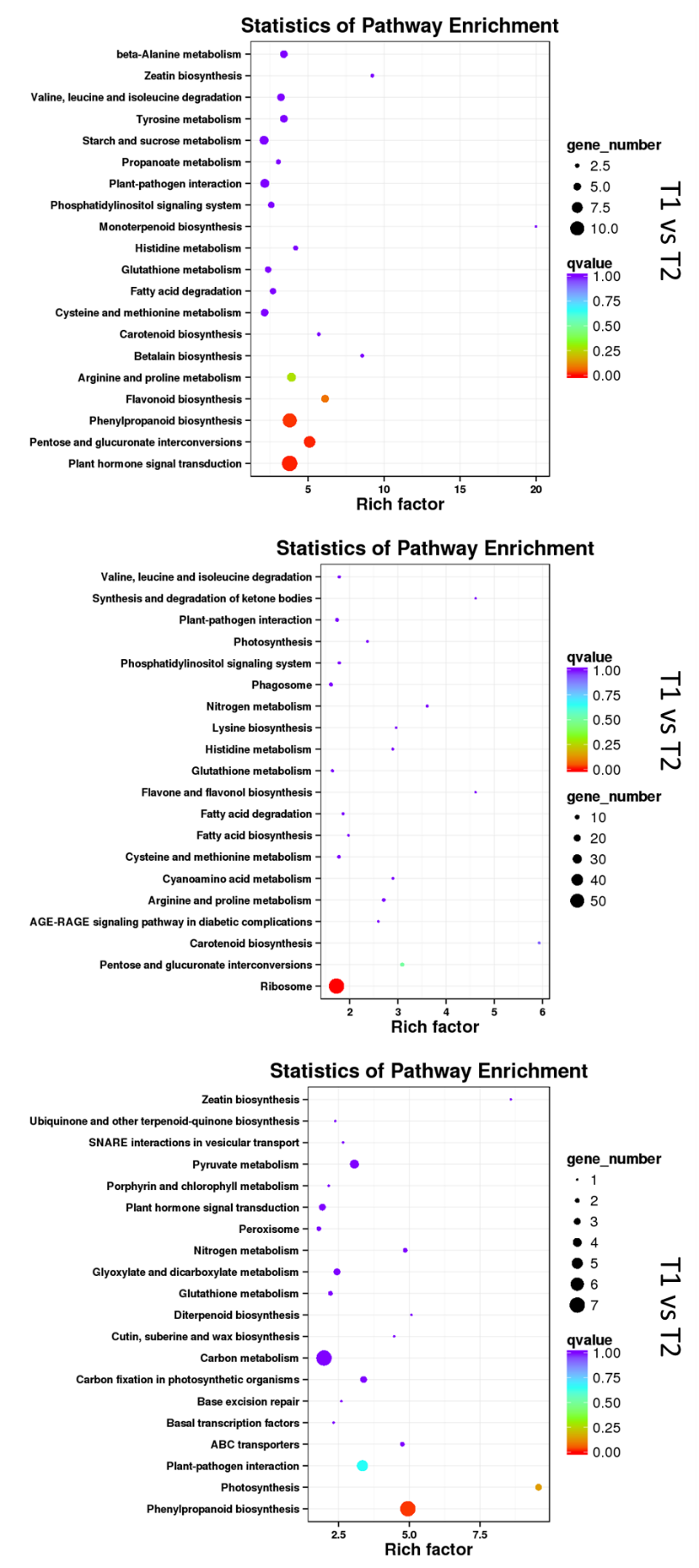


**Supplementary Figure 2.** KEGG pathway enrichment based on differentially expressed proteins between treatments.


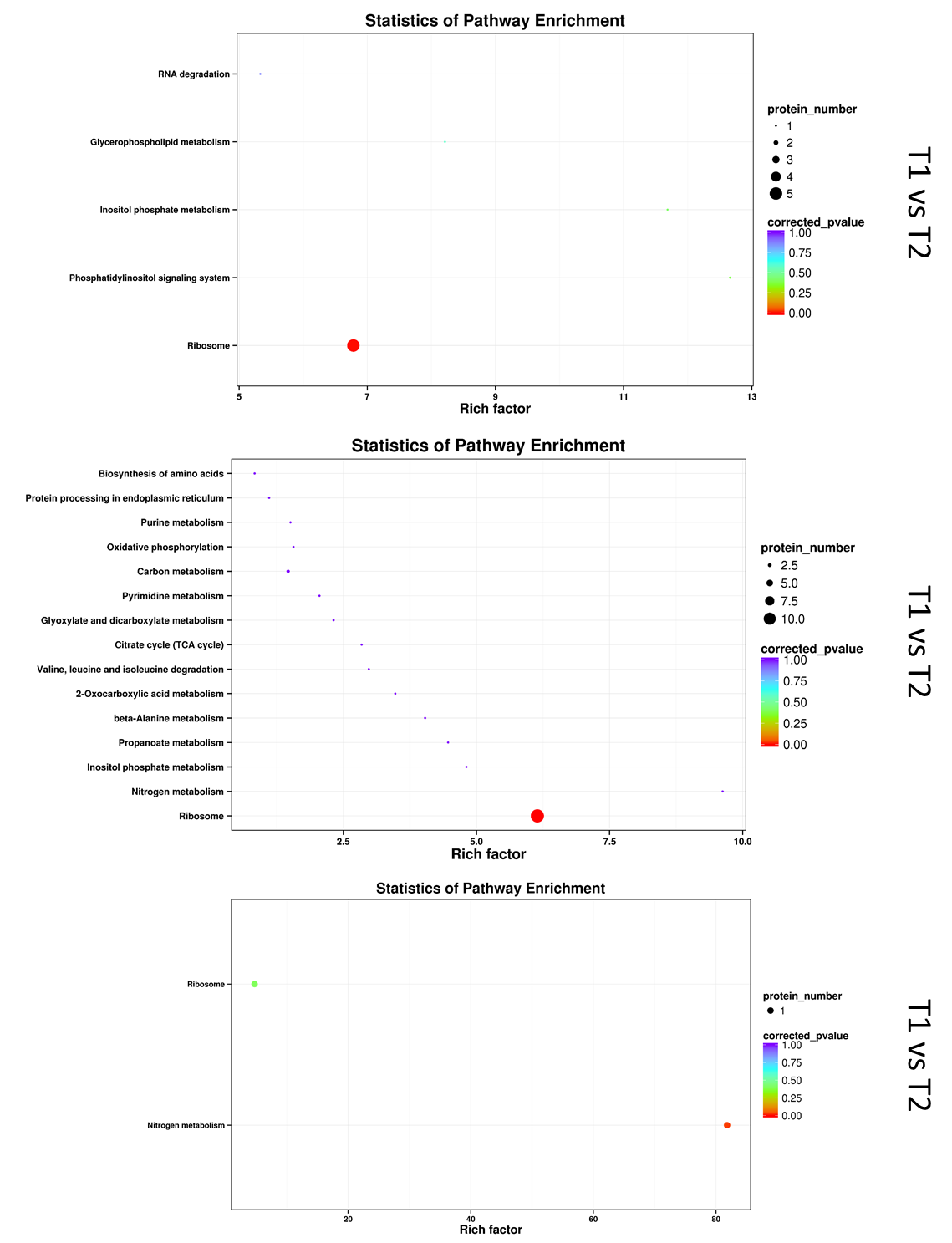

Supplement: Supplementary file 1 [file ijms-21-07548-s001.zip › Supps final/R1 Supplementary Figures.docx]
